# Robotics total knee arthroplasty: is an image-based the only solution?

**DOI:** 10.3389/fsurg.2025.1732887

**Published:** 2026-01-22

**Authors:** Alessandro El Motassime, Lorenzo Fulli, Luca Andriollo, Corrado Ciatti, Rudy Sangaletti, Francesco Benazzo, Stefano Marco Paolo Rossi

**Affiliations:** 1 Ortopedia e Traumatologia, Fondazione Poliambulanza, Brescia, Italy; 2 Dipartimento di Scienze Geriatriche e Ortopediche, Università Cattolica del Sacro Cuore, Rome, Italy; 3 Department of Orthopedics, Ageing and Rheumatological Sciences, Fondazione Policlinico Universitario A. Gemelli IRCCS, Rome, Italy; 4 Artificial Intelligence Center, Alma Mater Europaea University, Vienna, Austria; 5 Dipartimento di Ortopedia e Traumatologia, Ospedale Guglielmo da Saliceto, Piacenza, Italy; 6 IUSS Istituto Universitario di Studi Superiori, Pavia, Italy; 7 Department of Life Science, Health, and Health Professions, Università Degli Studi Link, Link Campus University, Roma, Italy; 8 OrtoTrauma Department, Arthroplasty Team, ASST Grande Ospedale Metropolitano Niguarda, Milano, Italy

**Keywords:** arthroplasty, knee, robot, imageless TKA, TKA

## Abstract

Robotic-assisted total knee arthroplasty (TKA) significantly enhances surgical precision and alignment accuracy. While traditional robotic systems rely on preoperative imaging, imageless technology has emerged as a viable alternative, leading to a reduction in costs, radiation exposure, and logistical challenges. This narrative review aims to evaluate the efficacy and reliability of imageless robotic-assisted TKA, specifically assessing its accuracy in component positioning, functional outcomes, and potential advantages over image-based systems. A review of current literature was conducted, comparing imageless robotic TKA with both manual and image-based techniques. The key parameters analyzed include alignment precision, joint line restoration, patient-reported outcomes, and complication rates. Notably, imageless robotic-assisted TKA demonstrated alignment accuracy and functional outcomes comparable to those of image-based systems, while providing enhancements in workflow efficiency and the elimination of radiation exposure. Although a moderate learning curve was observed, no significant differences in patient satisfaction or clinical performance were recorded. Overall, imageless robotic-assisted TKA represents a safe and effective alternative to image-based systems, achieving comparable surgical precision with additional economic and practical benefits. Further research is required to confirm long-term outcomes and to optimize intraoperative guidance strategies.

## 1 Introduction

Knee osteoarthritis (OA) is one of the most common joint disorders globally, especially among older adults. As life expectancy increases and population ages, the incidence of knee OA is rising, resulting in a greater need for effective treatments. Total knee arthroplasty (TKA) is considered the gold standard treatment for end-stage knee OA, aimed at relieving pain and restoring function when conservative methods, such as physical therapy or intra-articular injections, do not provide adequate relief (1).

**Table 1:** Summary on the main findings regarding different TKA techniques.

Feature	Manual TKA	Image-based RA-TKA	Imageless RA-TKA
Preoperative requirement	No imaging beyond standard radiographs	CT or MRI to generate 3D model	No advanced imaging; intraoperative mapping
Data acquisition	Mechanical jigs and anatomic referencing	Preoperative 3D imaging + intraoperative verification	Real-time intraoperative landmark registration
Bone resection execution	Surgeon-guided manually	Robot executes/constrains according to CT/MRI plan	Robot executes/constrains based on intraoperative plan
Alignment accuracy	Most variable; higher outlier rates	High accuracy with low outlier frequency	Comparable accuracy to image-based; fewer outliers than manual
Soft-tissue balancing	Subjective, surgeon-dependent	Enhanced via preoperative 3D planning	Dynamic intraoperative balancing with kinematic data
Radiation exposure	None	Yes (CT-based)	None
Workflow efficiency	Highest preoperative efficiency	Preoperative planning time required	No preoperative imaging; streamlined workflow
Cost considerations	Lowest	Higher (imaging + planning)	Lower than CT/MRI-based robotic systems
Learning curve	Established	Moderate (7–41 cases in literature)	Shorter (≈6–19 cases)
Functional outcomes	Good but variable	Improved consistency	Comparable to image-based; improved early satisfaction in some studies

Although TKA is considered a gold standard treatment, approximately 15%–20% of patients remain dissatisfied. Both patient-specific factors and surgeon-controlled variables play a role in patient-reported outcome measures and satisfaction after TKA (2).

While conventional TKA (CTKA) techniques have demonstrated long-term success, challenges remain in achieving precise component alignment and optimal joint kinematics. Improvements in implant design, surgical techniques, and perioperative care have enhanced patient outcomes over the years. However, challenges such as variability in alignment, soft tissue balancing, and implant positioning still persist. These factors are crucial because they directly affect postoperative function, pain relief, and the longevity of the implants (3, 4).

Computer assisted technologies in TKA have revolutionized surgical techniques by overcoming the limitations of traditional manual methods. These systems are designed to enhance surgical precision, improve the consistency of alignment, and provide better balancing of the knee joint (5–9).

Computer-assisted technologies in TKA encompass both navigation-assisted (NATKA) and robotic-assisted approaches, each designed to enhance the precision of bone resections and implant positioning.

NATKA provides real-time intraoperative feedback on alignment, bone cuts, and component placement, functioning as a guidance system rather than directly executing the resections. It may rely on preoperative imaging (image-based systems) or exclusively on intraoperative anatomical registration (imageless systems).

Robotic-assisted TKA (RATKA) further advances this concept by allowing the robotic platform to perform or constrain bone resections according to a predefined plan (10).

The systems available on the market can be: active systems in these, the surgeon positions the robotic arm near the patient, and the robot autonomously performs the surgery; haptic systems here, the surgeon controls the instrument by pressing a “go” button, while the robot ensures that the movement stays within pre-determined boundaries; collaborative systems, in this model, the surgeon remains in charge of the procedure and works alongside a smart robotic tool (11).

Robotic systems can be categorized into two types: “closed” platforms and “open” platforms. Closed platforms are designed to work with a specific implant only, while open platforms offer flexibility, allowing the use of various implants and designs based on the surgeon's preferences or the patient's needs (12).

Traditionally, RATKA has relied on image-based systems, which require preoperative imaging such as computed tomography (CT) or magnetic resonance imaging (MRI) or x-rays (10). These systems offer detailed three-dimensional reconstructions of the patient's anatomy, allowing for more accurate surgical planning and execution. These imaging techniques facilitate detailed preoperative planning, ensuring precise bone resections and optimal implant placement (13–15). However, dependence on imaging also brings certain inherent drawbacks such as costs, radiation exposure, processing time, and the need to reproduce the landmarks required by the system (16, 17). These issues, along with the difficulties of incorporating imaging data into surgical workflows, have led to a growing interest in alternative methods.

Image-free robotic systems have emerged as a promising and efficient solution in surgical procedures.

A special mention is due to the ROSA Knee System, which is currently the only system that provides surgeons with the option to use image-based techniques, requiring long leg 2D x-rays, or may be used imageless. Initial studies indicate that image-free systems may offer similar precision and clinical outcomes as their image-based counterparts (18, 19).

The question arises: Is an image-based robotic approach truly essential for robotic TKA, or can imageless technologies provide similar or even better outcomes while overcoming the logistical and economic challenges associated with imaging? This narrative review aims to comprehensively compare these two approaches, evaluating their clinical efficacy, patient safety, economic impact, and practical feasibility. By doing so, we hope to clarify the future direction of robotic TKA and contribute to the broader discussion on optimizing care for patients with knee osteoarthritis in the context of precision medicine (Table 1).

## 2 Principles of imageless technology

Imageless RATKA systems rely on intraoperative data acquisition to map the patient's anatomy in real time. These systems use advanced tracking technologies, including optical sensors and intraoperative registration, to create a virtual model of the knee (20). By mapping anatomical landmarks and assessing soft tissue dynamics during surgery, imageless systems enable surgeons to make precise adjustments to component positioning and ligament balancing. This approach eliminates the need for preoperative imaging, reducing radiation exposure, cost, and logistical complexities associated with CT or MRI.

## 3 Advantages of imageless technology

Imageless systems have numerous benefits that address most challenges in RATKA:

Cost Efficiency: eliminating the need for preoperative imaging imageless systems significantly reduces the financial burden on healthcare providers and patients
Radiation-Free Workflow: compared to CT-based systems, imageless technology completely removes patient exposure to ionizing radiation.
Dynamic Adjustments: real-time intraoperative feedback facilitates the personalization of surgical planning, meticulously tailored to the individual patient's unique anatomy and biomechanics.
Accessibility: a simplified workflow enhances the replicability of these systems across different clinical settings, including those with restricted access to advanced imaging modalities.

## 4 Outcomes

### 4.1 Alignment accuracy

Multiple studies have robustly demonstrated that imageless RATKA consistently achieves great precision in alignment. Seidenstein et al. compared bone resection and alignment in RATKA without imaging to traditional methods. The robotic group showed better accuracy in bone resection angles (*p* < 0.05), with all angles below 0.6° (SD < 0.4°), except the femur flexion/extension angle under 1.3° (SD = 1°). Limb alignment, measured by the Hip-Knee-Ankle (HKA) angle, also had accuracy and SD below 1° in the robotic group. Accuracy in bone resection was superior in the robotic group for both posterior femoral and proximal tibial levels, all below 0.7 mm accuracy (SD < 0.7 mm), and significant (*p* < 0.05). The study found that RATKA achieved better HKA alignment than conventional methods, with 100% of robotic cases within 3° of the target HKA, while only 75% of conventional cases met this standard. Moreover, 93% of robotic cases were within 2° of the target compared to 60% in the conventional group (21).

Furthermore Parratte et al. examined the ROSA Knee System, focusing on bone resection accuracy and component alignment using an imageless solution. They conducted the study on fifteen frozen cadaveric specimens (30 knees) with cuts guided by intraoperative mapping, omitting preoperative imaging. Results were validated against the computer-assisted navigation standard (ORTHOsoft, Zimmer Biomet). Mean differences between planned and achieved angles were below 1° for all cases except femoral sagittal flexion, which deviated by −0.95° (*p* < 0.001). The global HKA axis deviation was −0.03° ± 0.87°, with 73% within 1° and 97% within 2° of planned alignment. Resection thickness deviations were under 1 mm, except for the medial tibial plateau (0.66 mm, *p* < 0.001) and the medial distal femoral condyle (0.35 mm, *p* = 0.03) (22).

The research conducted by Doan et al. in 2022 evaluates the precision of an image-free RATKA system, comparing it to conventional instrumentation utilizing a cadaveric model. In this study, 40 cadaveric specimens were employed, whereby five orthopedic surgeons performed bilateral TKAs. One knee received robotic-assisted surgery utilizing the VELYS Robotic-Assisted System, while the contralateral knee was treated with conventional instrumentation. Pre-operative and post-operative CT scans, in conjunction with optical scans, were employed to assess implant alignment and resection accuracy. The results indicated a superior accuracy of the robotic-assisted system in femoral coronal alignment (0.63° ± 0.50° vs. 1.39° ± 0.95°, *p* < .001), femoral sagittal alignment (1.21° ± 0.90° vs. 3.27° ± 2.51°, *p* < .001), and tibial coronal alignment (0.93° ± 0.72° vs. 1.65° ± 1.29°, *p* = .001) in comparison to conventional methods. Moreover, the robotic-assisted cohort demonstrated a lower incidence of outliers (>3° errors) across all alignment metrics. The study concludes that image-free RATKA enhances the accuracy of implant alignment and diminishes alignment outliers, thereby substantiating its potential clinical advantages over traditional techniques (23).

A 2024 study by Edelstein et al. examined 61 patients undergoing RATKA with OMNIBotics, focusing on CPAK parameters like medial proximal tibial angle (MPTA) and lateral distal femoral angle (LDFA). It assessed these parameters via full-leg radiographs (LLR) and robotic navigation, based on hypotheses of 2 mm cartilage wear (Navlit) and an optimized wear hypothesis (Navopt). Comparisons included MPTA, LDFA, joint line obliquity, and HKA angles between robotic and LLR measurements. Results showed no significant mean differences in CPAK parameters: LLR vs. Navlit (delta <0.6°, *P* > 0.1) and Navopt (delta <0.1°, *P* > 0.83), with mean absolute errors: LDFA 1.4°, MPTA 2.0°, JLO 2.1°, and anatomic HKA 2.7°. Navlit categorized 88% of knees as in LLR, while Navopt reached 94%. Bland-Altman analyses indicated 95% and 91.8% agreement between LLR and Navlit/Navopt, confirming robotic navigation as a reliable alternative for CPAK parameters in TKA (24).

Schrednitzki et al. conducted a retrospective analysis comparing imageless robotic-assisted and conventional TKA. Their findings show that the robotic technique yields more accurate results. In their study, the mean difference between planned and measured axes was 1.01° ± 0.08° for RATKA, while the conventional group had a mean deviation of 2.05° ± 0.11°, which was statistically significant (*p* < 0.001). Robotic procedures demonstrated high precision in bone resections, with significant variation only at the distal medial femur and both posterior femoral condyles. All other discrepancies were ≤2 mm, averaging 0.21 mm. They concluded that the imageless pathway effectively preserves coronal alignment and bone resections, with accuracy comparable to other market systems (25).

Similarly, Bollars et al. conducted a trial comparing implant placement accuracy in TKA using NAVIO, an imageless robotic system, and conventional instruments. The study evaluated alignment outcomes through preoperative and postoperative CT scans. Results showed the imageless RATKA group had fewer alignment outliers (5.8% vs. 24.4%, *p* < 0.001). RATKA also had better femoral component alignment with a mean varus angle of 1.3° ± 1.7°, while CTKA had a mean valgus angle of −0.1° ± 1.9° (*p* = 0.005). The posterior tibial slope was measured more accurately in RATKA (1.4° ± 1.1° vs. 3.2° ± 1.8° in CTKA, *p* < 0.001). HKA axis alignment favored RATKA with a mean deviation of 0.4° ± 2.1° compared to −1.2° ± 2.1° in CTKA (*p* = 0.009). This study shows that imageless robotic systems improve accuracy in implant positioning and alignment over traditional methods, reducing imaging and logistical burdens while ensuring surgical precision (20).

The 2024 study by Mayne et al. examined the first 100 TKA surgeries using the ROSA system to evaluate prosthetic component positioning, focusing on joint line height, patellar height, and posterior condylar offset (PCO). Both imaging and non-imaging robotic systems were used, comparing joint line height, patellar height, and PCO by analyzing pre- and post-operative radiographs of patients averaging 70 years old (49–95 range). Mean variations were 0.2 mm for joint line height, 0.1 mm for patellar height (Insall-Salvati ratio), and 0.2 mm for PCO. No significant differences were noted between image-based and imageless robotic systems for these parameters (26).

Alton et al. conducted a study evaluating the VELYS Robotic-Assisted Solution for TKA, comparing its accuracy and early clinical outcomes with those of manual instrumentation during the adoption phase. This Level II, multicenter, prospective, non-randomized 1:1 cohort study was carried out at five sites, enrolling 200 patients (100 in the robotic-assisted group and 100 in the manual group). The primary endpoint was a non-inferiority analysis of HKA alignment accuracy, with secondary measures including femoral and tibial component positioning. The results indicated that RATKA significantly improved alignment accuracy (mMDFA of 1.3° vs. 1.9°, *p* = 0.0026; mMPTA of 1.2° vs. 1.5°, *p* = 0.026 (27).

The research conducted by Hasegawa et al. in 2023 presents a comparative analysis regarding the accuracy of component positioning and early clinical outcomes in TKA utilizing an imageless hand-held robotic-assisted system (Navio, Smith & Nephew), in contrast to a conventional jig-based technique. A total of 80 patients participated in the study (40 in the robotic group and 40 in the manual group), and an analysis was performed on the HKA angle, component alignment, and clinical outcomes at one year following the procedure. The findings indicated that implantation errors in both coronal and sagittal alignments were significantly lower in the robotic group, with no outliers exceeding errors of 3 degrees observed, while the manual group exhibited greater variability (28).

Similarly, Adamska et al. conducted a randomized controlled trial to evaluate the clinical and radiographic outcomes of imageless RATKA using the NAVIO and CORI systems. The study included 215 patients with knee OA, comparing their results with those of CTKA. They found that Radiographic accuracy showed that femoral component rotational alignment was more precise in NAVIO (1.48 ± 1.117°) and CORI (1.33 ± 1.012°) than in CTKA (3.15 ± 1.216°), with a significant difference (*p* = 0.0013) (29).

Furthermore, Yee et al. compared surgical accuracy in robotic-assisted total knee arthroplasty using image-free (NAVIO/CORI) and image-based (MAKO) methods, focusing on alignment outcomes, component placement, and functional recovery in 166 knees (71 image-free, 95 image-based) with end-stage osteoarthritis. They found that while both systems were comparable in coronal alignment accuracy, the image-free approach showed better accuracy with fewer tibial slope deviations and alignment outliers. The image-free method dynamically adapts to patient anatomy and showed similar or greater accuracy in component alignment, particularly in sagittal alignment, suggesting it may be a cost-effective, efficient alternative in robotic knee arthroplasty (30).

Gamie et al. in 2024 conducted a retrospective study evaluating the accuracy of the ROSA imageless RATKA in comparison with CTKA. They compared each system's ability to restore joint biomechanics, focusing on joint line height (JLH) and posterior condylar offset (PCO). This study had 2 groups matched in age and sex, with 182 RATKA procedures and 144 CTKA procedures. The authors found that the ROSA knee robotic system can more accurately restore JLH and PCO compared to CTKA (31).

In a similar manner in 2024, Rajgor et al. compared the surgical accuracy of the ROSA imageless robotic system with the MAKO image-based system in TKA. They assessed each system's ability to restore joint biomechanics, focusing on JLH, patella height (PH), tibial slope (TS), and PCO. This retrospective review covered 100 consecutive TKAs, with 50 procedures using each system by two experienced surgeons. There was no significant difference in JLH, PCO, TS, or PH. Both systems effectively restored native joint biomechanics, showing no superiority in the evaluated parameters. This study highlights the comparable accuracy of the ROSA and MAKO systems in TKA component positioning (32).

### 4.2 Functional outcomes

As previously mentioned in 2025, Alton et al. conducted a multicenter prospective study comparing 100 TKA performed with the VELYS Robotic-Assisted Solution for TKA and 100 CTKA. The secondary measures of this study include patient reported outcome measures (PROMs), and adverse events. The results indicated that robotic-assisted TKA significantly improved total performance score (TPS) of 1.7° vs. 2.8°, *p* < 0.0001) At the 12-week mark, patients who underwent robotic-assisted surgery reported better pain relief and higher Forgotten Joint Scores. However, at the one-year follow-up, PROMs were comparable between the two groups (27).

Blum et al. conducted a prospective study investigating the relationship between expectation fulfillment and patient satisfaction in RATKA over a follow-up period of two years. A total of 106 patients underwent RATKA utilizing the OMNIBotics system, with expectation fulfillment at three and six months correlated with satisfaction at one and two years. Patient-reported outcomes, including the KOOS and KSS, were evaluated preoperatively and postoperatively at three months, six months, one year, and two years. The results indicated that higher levels of expectation fulfillment at three months and six months were significantly associated with increased satisfaction at one year (*p* = 0.0012) and two years (*p* = 0.0323). In comparison to the data from the FORCE-TJR national database, RATKA patients demonstrated significantly greater improvements in KOOS sub-scores pertaining to pain, symptoms, sports and recreation, and quality of life; however, the absolute postoperative scores were comparable. This study underscores the significance of early patient expectation fulfillment in enhancing long-term satisfaction and supports RATKA as a procedure that meets or surpasses standard-of-care benchmarks for patient-reported outcomes (33).

As previously stated, in 2023, Hasegawa et al. conducted a study comparing 40 RATKA procedures performed with Navio and 40 CTKA procedures. No significant differences were observed in postoperative ROM, Knee Society Scores, or the Forgotten Joint Score between the two groups. Although the RATKA procedure exhibited a longer operative time (169.7 min compared to 119.8 min, *p* < 0.001), it showcased superior accuracy in bone preparation and component positioning. The study concludes that, while RATKA enhances surgical precision and reduces alignment errors, the early clinical outcomes remain comparable to those of conventional techniques (28).

As previously stated, Adamska et al. conducted a randomized controlled trial to evaluate RATKA performed with the NAVIO and CORI systems, comparing their results with those of CTKA. They found that postoperative Knee Injury and Osteoarthritis Outcome Study (KOOS) scores were highest in the NAVIO group (87.05 ± 7.74), followed by the CORI group (85.59 ± 8.03) and the CTKA group (81.76 ± 8.95), with significant differences (*p* = 0.0001). Improvements in ROM and VAS scores were similar across all groups (29).

As previously acknowledged, in 2024, Yee et al. conducted a study comparing RATKA performed using imageless systems (NAVIO/CORI) and image-based systems (MAKO) methods. At 12 months, functional outcomes were similar, but Knee Society Scores were slightly higher for the image-free group (95 vs. 92, *p* = 0.020) (30).

### 4.3 Operative time

In 2021, Savov et al. conducted a study concerning the learning curve and surgical time of RATKA performed with Navio compared with CTKA. The study demonstrated that the initial learning curve encompassed 11 cases. Subsequently, once this learning phase was complete, the operative times for imageless RATKA were comparable to those for the traditional technique, with RATKA averaging 69 min vs. 67 min for CTKA (*p* = 0.491) (34).

In a comparable study, Bosco et al. (2025) reported a mean surgical time of 72 min (±18.4) for RATKA performed with Navio system. Through segmented regression analysis of the learning curve, the authors observed that, although there was no statistically significant change in accuracy over time, operative duration decreased notably after the initial 11 cases (35).

In 2024, Burgio et al. conducted a multicenter retrospective study involving 118 RATKA performed on patients with knee OA. Despite extended surgical durations (121 ± 20.5 min) when compared to CTKA, RATKA did not demonstrate an elevated risk of PJI (36).

In the aforementioned study conducted by Alton et al., which evaluated the VELYS Robotic-Assisted Solution for TKA, the authors observed that initially, longer surgical durations were recorded; however, these durations improved with growing experience (27).

As acknowledged, in 2023 Adamska et al. conducted a randomized controlled trial comparing RATKA (Navio and CORI systems) and CTKA. They found that Robotic-assisted surgeries took longer: 44.5 min for NAVIO and 38.5 min for CORI (*p* = 0.003), but they resulted in less blood loss compared to CTKA: CTKA at 2.52 g/dL Hb, NAVIO at 1.74 g/dL Hb, and CORI at 1.51 g/dL Hb (*p* = 0.042) (29).

### 4.4 Complication rates

In 2024, Burgio et al. conducted a multicenter retrospective study that examined the incidence of early and delayed periprosthetic joint infection (PJI) in patients undergoing RATKA employing the NAVIO Surgical System between 2020 and 2023. A total of 118 patients were subjected to analysis, with a mean follow-up duration of 9.1 ± 3.9 months, aligning with the criteria established by the European Bone and Joint Infection Society (EBJIS). The findings revealed that no cases of early or delayed PJI were identified, as none of the patients fulfilled the EBJIS definitions of “Infection Likely” or “Infection Confirmed.” Two patients required revision surgery due to patellar maltracking and prosthetic loosening, while three patients underwent manipulation under anesthesia to address knee stiffness. The study concludes that RATKA is a secure technique, exhibiting no evidence of short-term PJI risk, thereby affirming its reliability in infection prevention (36).

Alton et al. in 2025, as previously acknowledged, conducted a multicenter prospective study evaluating the VELYS Robotic-Assisted Solution for TKA. The secondary measures include PROMs, and adverse events. The results indicated that RATKA significantly reduced the incidence of serious adverse events (6 in the robotic-assisted group vs. 16 in the manual group, *p* = 0.040) (27).

As previously noted in their prospective study conducted in 2021, Blum et al. included 106 RATKA procedures performed using the OMNIBotics system. Despite the absence of major complications or infections, two patients necessitated manipulation under anesthesia due to arthrofibrosis, representing 1.9% of the sample (33).

### 4.5 Cost analysis

Imageless RATKA incurs higher intraoperative costs than CTKA, primarily due to robotic system acquisition, maintenance, and disposables, with typical intraoperative costs around $10,295 per case vs. $9,999 for CTKA. Image-based RATKA is even more costly, as it requires preoperative CT imaging and has higher system expenses (37–40). For example, the case-related expenses at low-volume institutions, including CT imaging and equipment amortization, may surpass USD 70,000 and decrease to less than USD 4,000 per case at centers with high volume on account of fixed cost dissemination (41).

Despite these higher upfront costs, both imageless and image-based RATKA demonstrate reduced length of stay and lower post-acute care utilization compared to CTKA, resulting in lower 90-day episode-of-care costs. Imageless RATKA shows a 25% reduction in length of stay and a 57% reduction in opioid prescriptions, with 90-day episode-of-care costs approximately $2,090 lower than CTKA ($15,630 vs. $17,721). Image-based RATKA, while more expensive upfront, can achieve further reductions in length of stay and improved patient-reported outcomes, especially in high-volume centers, and may be cost-effective when annual procedure volume exceeds 49 cases (37, 39, 42, 43).

Operative time is longer for both robotic approaches compared to manual TKA, with image-based RATKA generally offering a modest reduction in surgical time compared to imageless RATKA. Complication rates are similar across all modalities, with robotic-assisted techniques (both imageless and image-based) showing slightly lower rates than CTKA, but no significant difference between imageless and image-based RATKA (37, 40, 44).

In summary, imageless RATKA is more costly than CTKA in terms of intraoperative expenses, but less costly than image-based RATKA. Both robotic approaches can achieve lower episode-of-care costs and improved value in high-volume settings, with cost-effectiveness driven by reductions in length of stay, opioid use, and post-acute care utilization.

Key findings are summarized as follows:

Alignment Accuracy: imageless systems demonstrate an accuracy in component alignment that is comparable to that of image-based systems, while exhibiting a reduced incidence of alignment outliers.
Functional Outcomes: postoperative improvements in functional scores, such as the Knee Society Score (KSS) and the Oxford Knee Score (OKS), are observed to be similar between RATKA utilizing imageless and image-based methodologies.
Operative Time: imageless systems may facilitate shorter durations for preoperative preparations; however, the overall surgical duration is frequently marginally longer when compared to CTKA procedures.
Complication Rates: the employment of imageless technology is associated with minimal complication rates, with various studies indicating comparable or enhanced safety profiles in relation to image-based techniques.

## 5 Challenges and limitations

Learning Curve: Surgeons transitioning to imageless systems may encounter an initial learning curve, particularly in the precise registration of anatomical landmarks.
Soft Tissue Management: Although imageless systems offer robust tools for alignment, achieving optimal ligament balancing may require additional expertise.
Limited Long-Term Data: Despite promising short-term and mid-term outcomes, further studies are required to validate the long-term durability of imageless RATKA.

Regarding learning curve several studies were conducted trying to assess its duration. Literature agrees that the learning curve varies ranging from 6 to 19 cases, varying depending on the system used, which is shorter than the learning curve for the image based which has been found to range from 7 to 41 cases (45).

## 6 Future directions

Progress in sensor design, artificial intelligence, and machine learning is anticipated to refine imageless robotic systems further. These advancements are expected to enhance the adaptability of these technologies to diverse surgical scenarios. Moreover, the incorporation of augmented reality (AR) tools into surgical workflows may enable more precise planning and streamline postoperative rehabilitation protocols. The integration of augmented reality and machine learning into robotic-assisted knee arthroplasty holds great promise by enhancing surgical precision, personalizing implant positioning, and accurately predicting patient outcomes using real-time data from wearable sensors and preoperative metrics. These advanced technologies can further optimize surgical results and patient satisfaction, marking a significant leap forward in orthopedic care (11, 46, 47).

Furthermore, imageless RATKA may also prove valuable in the context of revision knee arthroplasty, as suggested by emerging evidence. Imageless RATKA is considered valuable in revision total knee arthroplasty because it enables accurate intraoperative mapping and execution of bone resections and implant positioning without relying on preoperative imaging, which is often limited or distorted in the revision setting due to existing implants, hardware, or bone loss. This approach allows for real-time registration of anatomical landmarks, facilitating restoration of the joint line and optimal alignment even when conventional bony references are compromised. As research in this area advances, ongoing evaluation of the long-term advantages and possible limitations of imageless robotic systems will be crucial to determine their place as a standard of care in revision TKA (4, 48–50).

This, in turn, indicates that large-scale, multi-center clinical trials will be necessary in order to determine evidence-based guidelines and confirm the long-term reliability of imageless RATKA. The clearly defined and balanced patient cohorts (stratified based on age, BMI, preoperative alignment, and deformity severity) should be included in such a study, along with standardized alignment strategy and implant selection to reduce confounding. Principal outcomes to be measured will include alignment accuracy, operative time, complication and revision rates, and validated functional measures assessed at uniform follow-up points. Specifically, the functional measures will be according to well-established scoring systems, such as KOOS, WOMAC, and OKS, at 6 weeks, 3 months, 1 year, and mid- to long-term. Well-powered, protocol-harmonized multicenter designs will be important for defining whether imageless RATKA confers measurable advantages over image-based systems or conventional TKA.

## 7 Conclusions

Imageless RATKA has become a reliable and effective option for enhancing surgical precision and outcomes. This approach not only simplifies logistical processes but also reduces patient exposure to radiation. Current evidence shows that imageless robotic systems can achieve results in radiography and functionality that are comparable, if not superior, to those obtained through image-based and manual techniques. This is especially evident in the optimization of alignment and the reduction of complications. Additionally, the decreased requirement for preoperative imaging not only lowers costs but also increases accessibility, allowing a wider range of surgical settings to adopt this technology. Although longer surgical times compared to manual techniques are a factor to consider, the advantages of improved precision and reduced blood loss during surgery highlight the value of imageless robotic systems. As the field continues to evolve, additional studies are necessary to assess long-term clinical and economic outcomes. Nonetheless, imageless robotic-assisted TKA represents a reliable and promising advancement in knee surgery, supporting the goals of precision medicine and personalized patient care.
